# Pulse pressure variation and stroke volume variation during increased intra-abdominal pressure: an experimental study

**DOI:** 10.1186/cc9980

**Published:** 2011-01-19

**Authors:** Didier Jacques, Karim Bendjelid, Serge Duperret, Joëlle Colling, Vincent Piriou, Jean-Paul Viale

**Affiliations:** 1 Department of Emergency and Medical Intensive Care, Centre Hospitalier Lyon Sud, 165 Chemin du Grand Revoyet, 69495 Pierre Bénite Cedex, France; 2 Department of Anesthesiology, Pharmacology and Intensive Care, Intensive Care Service, Geneva University Hospitals, Rue Gabrielle-Perret-Gentil 4, 1211, Geneva, Switzerland; 3 Department of Anesthesiology and Intensive Care, Groupe Hospitalier Nord, Hospices Civils de Lyon, 103 Grande-Rue de la Croix-Rousse, 69317 Lyon Cedex 04, France; 4 Department of Anesthesiology and Intensive Care, Centre Hospitalier Lyon Sud, 165 Chemin du Grand Revoyet, 69495 Pierre Bénite Cedex, France; 5 Inserm, EA 4173 ERI 22, Laboratory of Physiology, University Claude Bernard Lyon 1, 8 avenue Rockefeller, 69008 Lyon, France

## Abstract

**Introduction:**

The aim of this study was to evaluate dynamic indices of fluid responsiveness in a model of intra-abdominal hypertension.

**Methods:**

Nine mechanically-ventilated pigs underwent increased intra-abdominal pressure (IAP) by abdominal banding up to 30 mmHg and then fluid loading (FL) at this IAP. The same protocol was carried out in the same animals made hypovolemic by blood withdrawal. In both volemic conditions, dynamic indices of preload dependence were measured at baseline IAP, at 30 mmHg of IAP, and after FL. Dynamic indices involved respiratory variations in stroke volume (SVV), pulse pressure (PPV), and systolic pressure (SPV, %SPV and Δdown). Stroke volume (SV) was measured using an ultrasound transit-time flow probe placed around the aortic root. Pigs were considered to be fluid responders if their SV increased by 15% or more with FL. Indices of fluid responsiveness were compared with a Mann-Whitney U test. Then, receiver operating characteristic (ROC) curves were generated for these parameters, allowing determination of the cut-off values by using Youden's method.

**Results:**

Five animals before blood withdrawal and all animals after blood withdrawal were fluid responders. Before FL, SVV (78 ± 19 vs 42 ± 17%), PPV (64 ± 18 vs 37 ± 15%), SPV (24 ± 5 vs 18 ± 3 mmHg), %SPV (24 ± 4 vs 17 ± 3%) and Δdown (13 ± 5 vs 6 ± 4 mmHg) were higher in responders than in non-responders (*P *< 0.05). Areas under ROC curves were 0.93 (95% confidence interval: 0.80 to 1.06), 0.89 (0.70 to 1.07), 0.90 (0.74 to 1.05), 0.92 (0.78 to 1.06), and 0.86 (0.67 to 1.06), respectively. Threshold values discriminating responders and non-responders were 67% for SVV and 41% for PPV.

**Conclusions:**

In intra-abdominal hypertension, respiratory variations in stroke volume and arterial pressure remain indicative of fluid responsiveness, even if threshold values identifying responders and non-responders might be higher than during normal intra-abdominal pressure. Further studies are required in humans to determine these thresholds in intra-abdominal hypertension.

## Introduction

Intra-abdominal pressure (IAP) is frequently increased in critically ill patients [1], and a sustained intra-abdominal hypertension (IAH) has been claimed to induce multiple organ failure and death [2]. In critically ill patients with acute circulatory failure due to IAH or other causes, fluid resuscitation could be indicated in order to increase cardiac output. However, any unnecessary volume loading has been shown to worsen the abdominal compartment syndrome (ACS) [3]. Therefore, dynamic indices of fluid responsiveness could be of value in this setting. Indeed, dynamic indices of fluid responsiveness relying on respiratory variations in arterial pressure or stroke volume have been developed in hypovolemic or septic settings [4-10]. Pulse pressure variation (PPV) and stroke volume variation (SVV) have been proved to be more reliable than static indices of preload such as right atrial pressure (RAP) or pulmonary capillary wedge pressure (PCWP). However, the predictive value of these dynamic indices in patients with IAH is unclear, as IAH affects respiratory variation in arterial pressure or stroke volume [11]. Recently, in an animal study, PPV proved to be predictive of fluid responsiveness during IAH, whereas, surprisingly, SVV was not [12]. In this study, the value of SVV was derived from pulse contour analysis, and could be, therefore, questionable. The purpose of our study was to evaluate the effects of IAH on indices of fluid responsiveness using aortic ultrasonic flow probe to measure SVV. We studied mechanically ventilated healthy pigs submitted to increased IAP and fluid loading (FL) before and after blood withdrawal.

## Materials and methods

### Animals and anesthesia

The experiment was conducted in nine pigs (weight 25 to 30 kg) according to the guidelines of the animal care committee of Claude Bernard University (Lyon, France). Animals were premedicated with ketamine (15 mg/kg) and were anesthetized with an injection of propofol (1 mg/kg) followed by continuous infusion of propofol (100 μg/kg/minute) and sufentanil (1 μg/kg/h). After tracheal intubation, pigs were mechanically ventilated (Servo ventilator 900 C-Siemens-Elema AB, Solna, Sweden) in a volume-controlled mode with a FiO_2 _of 0.4, a respiratory rate of 18/minute, an inspiratory:expiratory ratio of 1:2, an end-expiratory pressure of 0 cmH_2_0 and a tidal volume set in order to maintain the end-expiratory partial pressure of CO_2 _within the normal range. This tidal volume was kept constant during the experiment (13 ± 1 ml/kg).

A fluid-filled catheter was inserted into a carotid artery to monitor arterial pressure. Another catheter was placed in an internal jugular vein for fluid and drug administration, and for measurement of RAP. A pulmonary artery catheter was inserted through the controlateral internal jugular vein into the pulmonary artery to measure pulmonary arterial pressure and PCWP. An 8-cm air-filled latex cylindral balloon (Marquette, Boissy St. Léger, France) was positioned in the peritoneal cavity via a stab wound to measure abdominal pressure. After medial sternotomy and longitudinal pericardiotomy, an ultrasound transit-time flow probe was placed around the aortic root (14 mm A series; Transonic System, Ithaca, NY, USA). The pericardium was then partially closed and suspended in a pericardial cradle. Thoracic drains were inserted in the pleural space. Pleural pressure (Ppl) was recorded with another air-filled balloon placed in the mediastinal pleural space before closing the chest (Marquette, Boissy St. Léger, France). A catheter measuring airway pressure (Paw) was put at the junction of the tracheal tube. Respiratory flow was measured with a pneumotachograph. All the pressure and flow signals were recorded with a multi-channel recording system (MP 100; Biopac System, Santa Barbara, CA, USA). Finally, the abdomen was banded with a Velcro belt maintained by three inextensible belts. A large inflatable balloon was placed between these belts to increase IAP in a progressive manner.

### Experimental protocol

**Figure 1:**
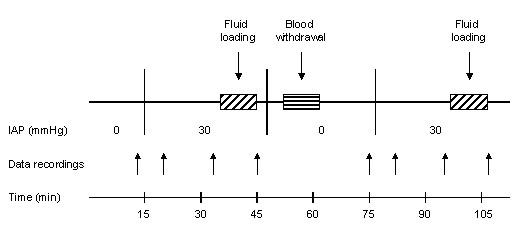
**Flow chart of the experimental protocol**.

After the surgical preparation, a 15-minute stabilization period was observed (Figure 1). Under steady-state anesthesia and normal IAP, circulatory and respiratory variables were recorded. Then, IAP was increased to 30 mmHg and maintained at this level, and data were recorded at this level of IAP. In order to perform FL, 500 ml of Ringer solution were infused for 10 minutes while IAP was kept at 30 mmHg. New data were collected just before and at the end of FL. The balloon was then deflated to decrease the IAP to its baseline level. Hypovolemia was created by blood withdrawal to a mean arterial pressure (MAP) of 60 mmHg. After a 15-minute stabilization, the same protocol and measurements were carried out at normal IAP and at IAP of 30 mmHg before another FL. So, before and after blood withdrawal, data were recorded under two IAP levels (0, 30 mmHg), and at IAP of 30 mmHg, before and after FL.

### Measurements and calculations

Heart rate (HR), MAP, mean RAP (RAPm), cardiac output, stroke volume (SV), mean Ppl, and mean IAP were analysed over five consecutive respiratory cycles. Maximal inspiratory and minimal expiratory Ppl were averaged from three consecutive breaths, as well as peak airway pressure (Peak Paw), inspiratory plateau pressure (Pplat), maximal inspiratory IAP, PCWP, respiratory variations in arterial systolic pressure (SPV), PPV and SVV. Transmural RAPm (RAPm-tm), transmural PCWP (PCWP-tm), pulmonary vascular resistance (PVR) and systemic vascular resistance (SVR) were calculated with the usual formula. The static compliance of the respiratory system (Crs) was calculated as the ratio of tidal volume to Pplat assuming that total positive end-expiratory pressure was equal to zero. The end-inspiratory transpulmonary pressure (Ptrans) was calculated as follows: Ptrans = Pplat - Ppl. The abdomino-thoracic pressure transmission index (ATI) was obtained using maximal inspiratory values of Ppl and IAP: ATI = (Ppl at IAP 30 - Ppl at IAP 0)/(IAP at IAP 30 - IAP at IAP 0). The inspiratory-induced Ppl increase (ΔPpl) was calculated as the difference between maximal inspiratory Ppl and minimal expiratory Ppl. SPV were split into its two components, Δup and Δdown, after comparison with systolic pressure recording during apnea. SPV was also expressed relatively to systolic pressure (SP) maximal value according to the following formula [8]: %SPV = (SPV/maximal SP) × 100. PPV and SVV were calculated as previously described [4,6].

### Statistical analysis

All values are shown as mean ± standard deviation (SD). Analysis of variance for repeated measures with Newman-Keuls *post-hoc *test was used to characterize the effects of IAP and volemia on the circulatory, respiratory and intra-abdominal parameters. We considered pigs to be fluid responders if their SV increased by 15% or more with FL. Indices of fluid responsiveness were compared with a Mann-Whitney U test. Then, receiver operating characteristic (ROC) curves were generated for these parameters. Identification of cut-off values was performed using the Youden's method. Finally, changes in SV with FL were compared to these indices by a simple linear regression analysis. Significance was considered for *P *< 0.05.

## Results

Before blood withdrawal, when IAP was raised to 30 mmHg, a significant decrease in SV was observed (Table 1). Non-transmural RAPm and RAPm-tm increased significantly, from 9.7 ± 3.5 to 17.6 ± 5.8 mmHg and from 7.8 ± 3.7 to 11.0 ± 5.6 mmHg, respectively (*P *< 0.05). A significant increase in PVR was also noticed. Non-transmural PCWP and PCWP-tm did not change significantly. Then, FL increased transmural filling pressures and SV globally. After return to baseline IAP, blood withdrawal induced a decrease in transmural filling pressures, SV and MAP, whereas HR increased, as expected. Then, after IAP was raised to 30 mmHg, SV did not change significantly, whereas MAP and SVR increased significantly. Two pigs had sustained arrhythmia during IAH and FL after blood withdrawal. Accordingly, complete data were available on seven pigs for this last part of the protocol. FL increased SV in all animals.

**Table 1:** Effects of alterations in IAP and volemia on circulatory and respiratory parameters

IAP (mmHg)	**0**^**†**^	**30**^**†**^	**30 before FL**^**†**^	**30 after FL**^**†**^
**HR (/minute)**				
Before blood withdrawal	105 ± 28	110 ± 19	121 ± 28	118 ± 23
After blood withdrawal	142 ± 28*	145 ± 20*	149 ± 27*	124 ± 19^♣^
**MAP (mmHg)**				
Before blood withdrawal	75.3 ± 9.5	80.5 ± 13.7	91.6 ± 9.0	102.7 ± 16.3
After blood withdrawal	50.0 ± 11.5*	64.9 ± 11.1^#^*	73.6 ± 8.8*	98.3 ± 25.5^♣^
**RAPm (mmHg)**				
Before blood withdrawal	9.7 ± 3.5	17.6 ± 5.8^#^	18.0 ± 5.9	24.3 ± 7.5^♣^
After blood withdrawal	7.2 ± 3.6*	13.1 ± 3.4^#^*	12.6 ± 3.3	20.6 ± 5.8^♣^
**RAPm-tm (mmHg)**				
Before blood withdrawal	7.8 ± 3.7	11.0 ± 5.6^#^	10.9 ± 6.7	16.8 ± 7.7^♣^
After blood withdrawal	4.9 ± 4.3*	6.4 ± 4.3*	7.2 ± 3.5	14.8 ± 5.2^♣^
**PCWP (mmHg)**				
Before blood withdrawal	10.0 ± 3.4	11.9 ± 3.9	11.9 ± 4.4	15.7 ± 3.5^♣^
After blood withdrawal	5.5 ± 2.8*	6.2 ± 2.7*	6.3 ± 2.7	12.2 ± 8.1
**PCWP-tm (mmHg)**				
Before blood withdrawal	10.1 ± 3.6	11.7 ± 4.5	11.0 ± 4.1	15.3 ± 4.3^♣^
After blood withdrawal	6.4 ± 2.5*	6.3 ± 2.6*	7.2 ± 2.7	14.5 ± 8.2
**SV (ml)**				
Before blood withdrawal	17.5 ± 4.3	14.0 ± 4.7^#^	13.7 ± 5.2	17.1 ± 4.4^♣^
After blood withdrawal	8.5 ± 2.9*	8.0 ± 3.2*	10.0 ± 2.5*	16.5 ± 3.3^♣^
**SVR (dynes.s.cm**^**-5**^**)**				
Before blood withdrawal	3,052 ± 872	3,653 ± 1401		
After blood withdrawal	3,194 ± 1354	4,440 ± 2158^#^		
**PVR (dynes.s.cm**^**-5**^**)**				
Before blood withdrawal	678 ± 230	1,383 ± 962^#^		
After blood withdrawal	1,147 ± 550	2,541 ± 2,239		
**Peak Paw (cmH**_**2**_**0)**				
Before blood withdrawal	27.7 ± 3.5	58.3 ± 8.0^#^		
After blood withdrawal	29.5 ± 3.3	56.5 ± 9.3^#^		
**Ptrans (cmH**_**2**_**0)**				
Before blood withdrawal	17.7 ± 3.8	28.8 ± 10.6^#^		
After blood withdrawal	18.4 ± 5.9	29.6 ± 15.0^#^		
**Crs (ml/cmH**_**2**_**0)**				
Before blood withdrawal	19.5 ± 3.2	7.3 ± 0.9^#^		
After blood withdrawal	20.3 ± 2.6	7.7 ± 0.9^#^		
**ΔPpl (mmHg)**				
Before blood withdrawal	4.4 ± 2.3	18.1 ± 10.7^#^		
After blood withdrawal	5.9 ± 4.4	17.7 ± 11.3^#^		
**IAPm (mmHg)**				
Before blood withdrawal	3.1 ± 2.3	30.3 ± 3.7^#^		
After blood withdrawal	3.1 ± 2.2	31.3 ± 1.7^#^		
**ATI (%)**				
Before blood withdrawal		47 ± 29		
After blood withdrawal		43 ± 31		

Definition of abbreviations: ATI, abdomino-thoracic pressure transmission index; Crs, static compliance of the respiratory system; ΔPpl, (maximal inspiratory pleural pressure - minimal expiratory pleural pressure); FL, fluid loading; HR, heart rate; IAPm, mean intra-abdominal pressure; MAP, mean arterial pressure; Paw, airway pressure; PCWP, pulmonary capillary wedge pressure; PCWP-tm, transmural pulmonary capillary wedge pressure; Ptrans, end-inspiratory transpulmonary pressure; PVR, pulmonary vascular resistance; RAPm, mean right atrial pressure; RAPm-tm, transmural mean right atrial pressure; SV, stroke volume; SVR, systemic vascular resistance.

^#^: *P *< 0.05 vs IAP 0; * : *P *< 0.05 vs before blood withdrawal; ^♣ ^: *P *< 0.05 vs IAP 30 before FL.

^**†**^: before blood withdrawal, *n *= 9 at IAP 0, 30, 30 before FL, and 30 after FL; after blood withdrawal, *n *= 9 at IAP 0 and 30, *n *= 7 at IAP 30 before FL and 30 after FL.

The increase in IAP induced significant changes in respiratory variables (Table 1). Peak Paw, Ptrans, and ΔPpl increased, whereas Crs decreased. ATI, which quantifies the amount of abdominal pressure transmitted to the thoracic compartment, was 47 ± 29% before blood withdrawal.

**Figure 2:**
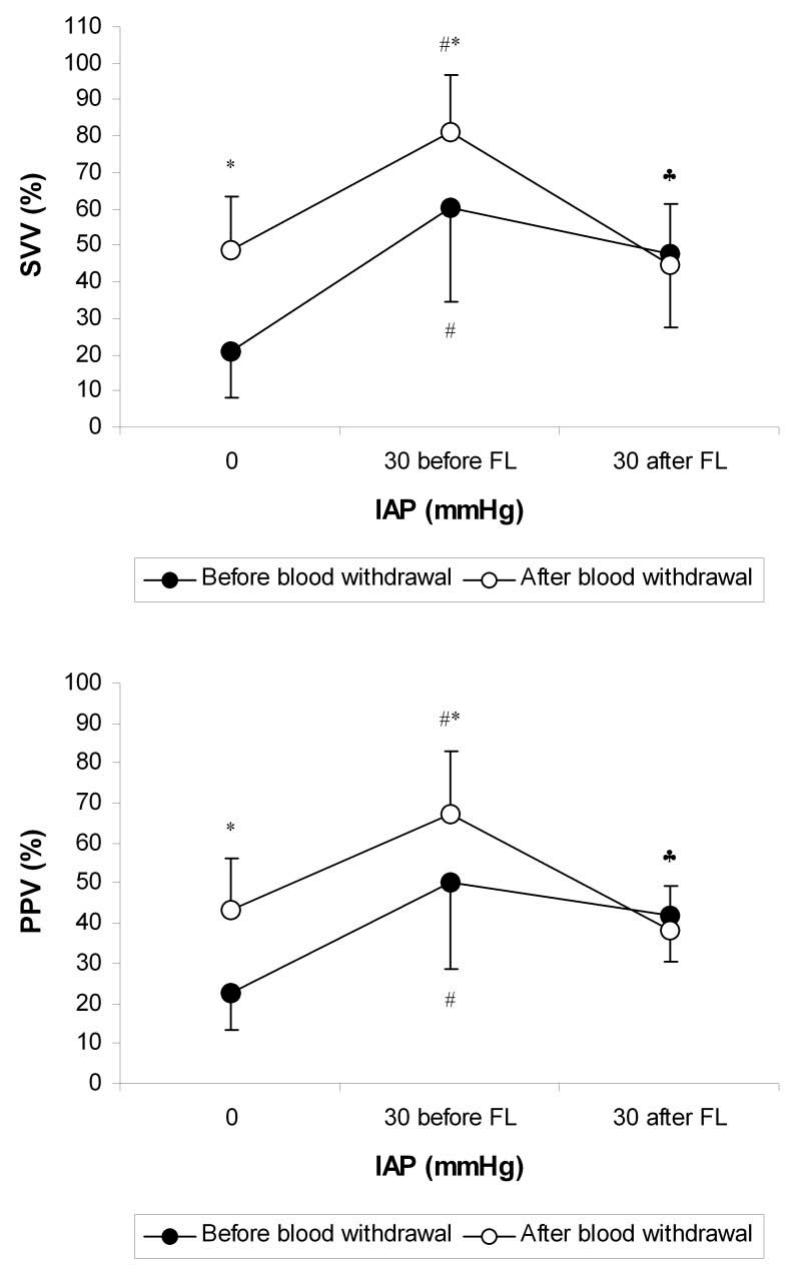
**Effects of alterations in IAP and volemia on SVV and PPV**. Definition of abbreviations: FL, fluid loading; IAP, intra-abdominal pressure; PPV, pulse pressure variation; SVV, stroke volume variation. ^# ^: *P *< 0.05 vs IAP 0; * : *P *< 0.05 vs before blood withdrawal; ^♣ ^: *P *< 0.05 vs IAP 30 before FL for the animals after blood withdrawal. Before blood withdrawal, *n *= 9 at IAP 0, 30 before FL and 30 after FL; after blood withdrawal, *n *= 9 at IAP 0, *n *= 7 at IAP 30 before FL and 30 after FL.

**Figure 3:**
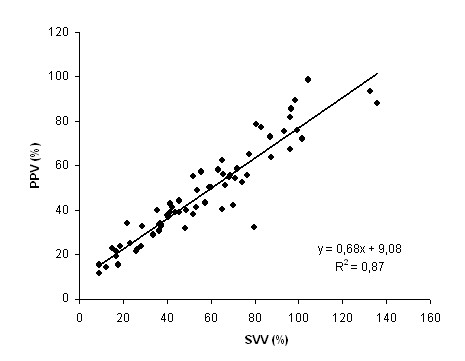
**Relation between PPV and SVV**. Definition of abbreviations: PPV, pulse pressure variation; SVV, stroke volume variation. The data were pooled from the different steps of the protocol (*n *= 67).

SVV and PPV increased with both IAH and blood withdrawal (Table 2 and Figure 2). They were strongly correlated (R^2 ^= 0.87, *P *< 0.0001, Figure 3). SPV also increased with both IAH and blood withdrawal. Alterations in SPV with IAP were mainly due to Δup increase. SPV and Δup were correlated with ΔPpl (R^2 ^= 0.42 and 0.47 respectively, *P *< 0.0001), whereas no correlation was found between Δdown and ΔPpl (R^2 ^= 0.01, *P *> 0.10). Similarly, correlation between ΔPpl and PPV or SVV were weak (R^2 ^= 0.19 and 0.28 respectively, *P *< 0.005).

**Table 2:** Effects of alterations in IAP and volemia on dynamic indices of fluid responsiveness

IAP (mmHg)	**0**^**†**^	**30**^**†**^	**30 before FL**^**†**^	**30 after FL**^**†**^
**SVV (%)**				
Before blood withdrawal	21 ± 13	57 ± 26^#^	60 ± 26	48 ± 20
After blood withdrawal	49 ± 15*	99 ± 24^#^*	81 ± 16*	45 ± 17^♣^
**PPV (%)**				
Before blood withdrawal	23 ± 9	50 ± 23^#^	50 ± 22	42 ± 11
After blood withdrawal	43 ± 13*	68 ± 20^#^*	67 ± 16*	38 ± 11^♣^
**SPV (mmHg)**				
Before blood withdrawal	7 ± 3	21 ± 5^#^	23 ± 5	22 ± 6
After blood withdrawal	11 ± 5*	24 ± 6^#^	26 ± 4	22 ± 6^♣^
**%SPV (%)**				
Before blood withdrawal	8 ± 3	19 ± 5^#^	19 ± 4	17 ± 4
After blood withdrawal	15 ± 5*	25 ± 4^#^*	26 ± 3*	18 ± 5^♣^
Δ**up (mmHg)**				
Before blood withdrawal	2 ± 3	13 ± 4^#^	11 ± 3	13 ± 4
After blood withdrawal	0 ± 4	9 ± 5^#^	8 ± 6	13 ± 4
Δ**down (mmHg)**				
Before blood withdrawal	6 ± 5	8 ± 5	8 ± 5	7 ± 1
After blood withdrawal	12 ± 7*	16 ± 6*	16 ± 4*	9 ± 3^♣^

Definition of abbreviations: FL, fluid loading; PPV, pulse pressure variation; SPV, systolic pressure variation; %SPV, (SPV/maximal systolic pressure) × 100; SVV, stroke volume variation.

^#^: *P *< 0.05 vs IAP 0; * : *P *< 0.05 vs before blood withdrawal; ^♣ ^: *P *< 0.05 vs IAP 30 before FL.

^**†**^: before blood withdrawal, *n *= 9 at IAP 0, 30, 30 before FL, and 30 after FL; after blood withdrawal, *n *= 9 at IAP 0 and 30, *n *= 7 at IAP 30 before FL and 30 after FL.

**Figure 4:**
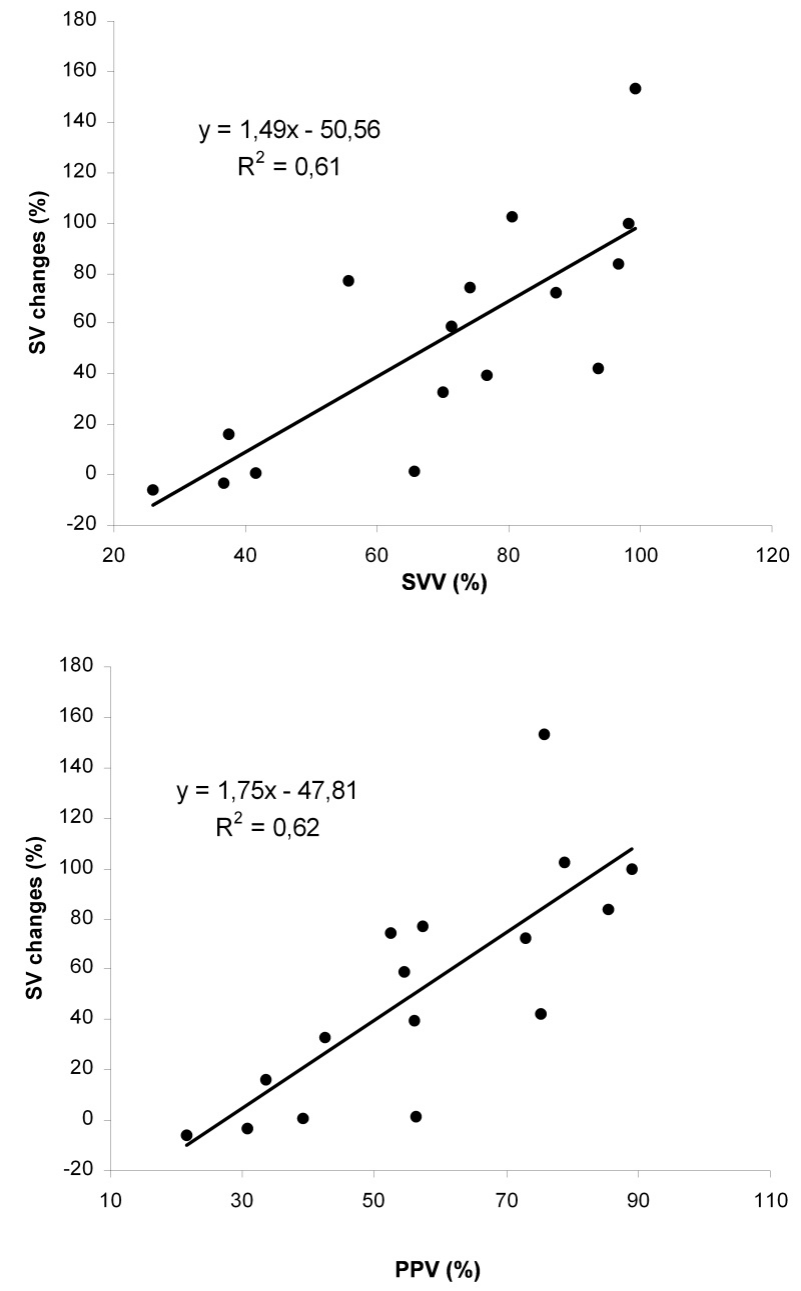
**Relation between changes in SV with FL and SVV or PPV before FL**. Definition of abbreviations: FL, fluid loading; PPV, pulse pressure variation; SV, stroke volume; SVV, stroke volume variation. FL was performed at intra-abdominal pressure of 30 mmHg in 9 pigs before blood withdrawal and 7 pigs after blood withdrawal (see also text).

Before blood withdrawal, FL did not change significantly SVV, PPV, SPV, %SPV, and Δdown (Table 2 and Figure 2). On the contrary, after blood withdrawal, SVV, PPV, SPV, %SPV, and Δdown decreased significantly with FL. In fact, before blood withdrawal, four pigs out of nine were non-fluid responders, whereas after blood withdrawal, all animals were fluid responders. Before FL, non-responders had lower SVV, PPV, SPV, %SPV, and Δdown at IAP of 30 mmHg than responders (Table 3). ROC curves data showed that areas for all these parameters were between 0.86 and 0.93 (Table 4). Threshold values discriminating non-responders and responders were quite high for SVV and PPV (67% and 41% respectively). Indeed, before blood withdrawal, SVV and PPV tended to be higher during IAH than during baseline IAP even in the non-responders: SVV increased from 21 ± 10% at baseline IAP to 42 ± 17% at IAP of 30 mmHg (*P *= 0.06), whereas PPV increased from 22 ± 9% to 37 ± 15% (*P *= 0.09). Changes in SV with FL were strongly correlated with pre-loading SVV and PPV values (R^2 ^= 0.61 and 0.62 respectively, *P *< 0.0005, Figure 4). They were less correlated with pre-loading %SPV and SPV values (R^2 ^= 0.43 and 0.26 respectively, *P *< 0.05), whereas no correlation was found with Δdown (R^2 ^= 0.23, *P *= 0.07).

**Table 3:** Indices of fluid responsiveness

	Non-Responders	Responders	*P*
**SVV (%)**	42 ± 17	78 ± 19	<0.05
**PPV (%)**	37 ± 15	64 ± 18	<0.05
**SPV (mmHg)**	18 ± 3	24 ± 5	<0.05
**%SPV (%)**	17 ± 3	24 ± 4	<0.05
Δ**down (mmHg)**	6 ± 4	13 ± 5	<0.05

Definition of abbreviations: FL, fluid loading; PPV, pulse pressure variation; SPV, systolic pressure variation; %SPV, (SPV/maximal systolic pressure) × 100; SVV, stroke volume variation.

FL was performed in nine pigs before blood withdrawal and seven pigs after blood withdrawal (see also text).

**Table 4:** ROC curves data

	Area	95% CI	*P*	Cut-off
**SVV (%)**	0.93	0.80 to 1.06	0.01	67
**PPV (%)**	0.89	0.70 to 1.07	0.03	41
**SPV (mmHg)**	0.90	0.74 to 1.05	0.02	22
**%SPV (%)**	0.92	0.78 to 1.06	0.02	22
Δ**down (mmHg)**	0.86	0.67 to 1.06	0.04	13

Definition of abbreviations: CI, confidence interval; FL, fluid loading; PPV, pulse pressure variation; SPV, systolic pressure variation; %SPV, (SPV/maximal systolic pressure) × 100; SVV, stroke volume variation.

## Discussion

In mechanically ventilated healthy pigs with IAH, the present study shows that SVV and PPV are still accurate indices of fluid responsiveness. However, threshold value discriminating responders and non-responders could be modified by IAH.

Fluid therapy is a major issue in critical care [13-16]. In mechanically ventilated patients, it relies more and more on dynamic indices of preload dependence, based on interactions between respiratory and circulatory functions [4-10,17-19]. However, the straightforward interpretation of these indices has been reassessed [20,21]. In a previous study, our group showed that in mechanically ventilated pigs, IAH affected respiratory variations in SV and arterial pressure [11,22]. As no FL was done, the fluid responsiveness predictive value of these indices remained questionable.

In the present study, circulatory changes induced by marked IAH before loading were similar to those described previously [23]. Indeed, SV decreased with IAH and hypovolemia. Before blood withdrawal, RAPm-tm, PVR, and Ptrans increased significantly with IAH, suggesting right ventricular afterload increase [24]. After blood withdrawal, MAP and SVR increased significantly with IAH, whereas SV decreased slightly, suggesting left ventricular afterload increase. When FL was performed, filling pressures and SV increased as a mean before or after blood withdrawal. However, before blood withdrawal, animals split into responders and non-responders, suggesting that relative hypovolemia was present during IAH in some animals. As expected, after blood withdrawal, all animals were fluid responders. In both cases, respiratory variations in SV and arterial pressure were more pronounced with IAH. However, they were still predictive of fluid responsiveness. SVV, PPV, SPV, %SPV and Δdown were significantly higher in responders. Among these indices, pre-loading SVV and PPV had the strongest correlation with changes in SV with loading. PPV could be more closely related to changes in SV than SPV because of its lesser dependence on IAH-induced Ppl swing. Indeed, PPV mostly reflected SVV. In this study, a PPV value of 41% separated responders and non-responders, suggesting that PPV threshold value identifying responders and non-responders could be higher in case of IAH. Recently, another animal study addressing the very same question but with another methodology was published [12,25]. In this study, an IAP around 25 mmHg also increased the threshold value for PPV from 11.5% to 20.5%. In humans, Mahjoub *et al. *[26,27] also noticed that among 41 mechanically ventilated patients with IAH and a PPV >12%, 10 (24.4%) were not fluid responders, suggesting that usual threshold value to predict fluid responsiveness could be altered by IAH. So, it seems that a high PPV value in IAH patients does not necessarily predict a positive fluid response. In our study, before blood withdrawal, PPV values at baseline IAP (23 ± 9%) were much higher than in humans, so that straight extrapolation of our threshold value of 41% to clinical practice could be hazardous. Nevertheless, even among non-responders, an increase in PPV and SVV was observed after increasing IAP. So, IAP could interfere with PPV and SVV independently of relative hypovolemia. Indeed, our results suggests an IAH-induced increase in right ventricular afterload, as already shown previously [28,29]. It could have resulted in an increase in respiratory variations in right ventricular SV, a situation where the predictive value of PPV to detect preload dependence has been questioned already [30,31]. Thus, the high PPV values observed during IAH could result from the addition of hypovolemia (which results in "preload dependence") and IAH-induced right ventricular afterload increase (which is "preload independent").

Conversely to the results of Renner *et al. *[12], we found that SVV is also predictive of fluid responsiveness in IAH. Renner *et al. *acquired SVV with the PiCCO system (Pulsion Medical Systems, Munich, Germany). This latter derives SV from pulse contour analysis of arterial femoral pressure, a derivation which could be biased in case of IAH and vascular constraint [12]. Indeed, an experimental study performed by the same group [32] supported this hypothesis as it showed that IAH affected the continuous cardiac output (and SV) measurement based on pulse contour analysis with the PiCCO system. The evoked explanation was the change in arterial impedance induced by IAH. In the present study, SVV was measured using an ultrasound transit-time flow probe placed around the aortic root. This measurement is probably less influenced by IAH. The strong correlation we found between PPV and SVV further reinforced the reliability of this SV measurement. Considering clinical practice where such a flow probe cannot be used, Doppler echocardiography could be useful during IAH. Indeed, SV can be assessed by recording flow in the left ventricular outflow tract and measuring velocity time integral (VTI). Furthermore, respiratory variation in VTI (or peak velocity as a surrogate) has already been shown to be predictive of fluid responsiveness at normal IAP [18,19]. As measuring SVV by Doppler echocardiography should be less biased by high IAP than pulse contour analysis of femoral pressure, respiratory variation in VTI (or peak velocity) could be predictive of preload dependence during IAH. Likewise, SVV from pulse contour analysis of radial pressure could be more reliable than pulse contour analysis of femoral pressure, as arterial radial impedance should be not affected by IAP.

This experimental study suffers from some limitations. First, as already mentioned, baseline PPV and SVV at IAP 0 were higher than in humans or in our previous experimental study [11]. High tidal volume could partly explain these findings. Furthermore, FL was performed at a high IAP level. Consequently, threshold values discriminating responders and non-responders cannot be directly extrapolated to clinical practice. As threshold values may be gradually increased by IAP, further studies are required in humans to determine specific thresholds within the four grades of IAH as defined by the International Conference of Experts on IAH and ACS [2]. Second, IAH was induced by abdominal compression without increase in abdominal volume as usually encountered in clinical conditions. Third, IAH duration was short, so that long-term effects of IAH could not be evaluated. Fourth, we included a small number of animals. However, it was similar to animal populations in numerous experimental studies [8,23,29,32]. Finally, we used healthy pigs. So, our results cannot be directly extrapolated to critically ill patients.

## Conclusions

Our findings suggest that in the presence of IAH, variations in arterial pressure or SV related to mechanical ventilation remain indices of fluid responsiveness. However, threshold values discriminating responders and non-responders might be increased. PPV and SVV seem more accurate than SPV. As different thresholds may be obtained at different IAP, further studies are needed in humans to determine specific thresholds within different IAP ranges.

## Key messages

• In this experimental study, variations in arterial pressure or SV related to mechanical ventilation remain indices of fluid responsiveness during IAH.

• PPV and SVV seem more accurate than SPV.

• Threshold values discriminating responders and non-responders might be higher than during normal IAP, so that a "supra normal" SVV or PPV does not necessarily mean fluid responsiveness.

• As thresholds may vary with IAP levels, further studies are needed in humans to determine specific thresholds within the different grades of IAH.

## Abbreviations

ACS: abdominal compartment syndrome; ATI: abdomino-thoracic pressure transmission index; Crs: static compliance of the respiratory system; Δdown: decrease in systolic arterial pressure during ventilation using the systolic pressure during apnea as reference; ΔPpl: (maximal inspiratory pleural pressure - minimal expiratory pleural pressure); Δup: increase in systolic arterial pressure during ventilation using the systolic pressure during apnea as reference; FL: fluid loading; HR: heart rate; IAH: intra-abdominal hypertension; IAP: intra-abdominal pressure; IAPm: mean intra-abdominal pressure; MAP: mean arterial pressure; Paw: airway pressure; PCWP: pulmonary capillary wedge pressure; PCWP-tm: transmural pulmonary capillary wedge pressure; Peak Paw: peak airway pressure; Ppl: pleural pressure; Pplat: inspiratory plateau pressure; PPV: pulse pressure variation; Ptrans: end-inspiratory transpulmonary pressure; PVR: pulmonary vascular resistance; RAP: right atrial pressure; RAPm: mean right atrial pressure; RAPm-tm: transmural mean right atrial pressure; ROC: receiver operating characteristic; SP: systolic pressure; SPV: systolic pressure variation; %SPV: systolic pressure variation during ventilation expressed relatively to systolic pressure maximal value; SV: stroke volume; SVR: systemic vascular resistance; SVV: stroke volume variation; VTI: velocity time integral.

## Competing interests

The authors declare that they have no competing interests.

## Authors' contributions

DJ participated in the design of the study and in the experiments, performed the data analysis and the statistical analysis, and drafted the manuscript. KB conceived the study, participated in the experiments, and helped to draft the manuscript. SD, JC and VP participated in the design of the study and in the experiments, and helped to draft the manuscript. JPV conceived the study, participated in the experiments, and helped to draft the manuscript. All authors read and approved the final manuscript.
